# Experimental imaging in orthotopic renal cell carcinoma xenograft models: comparative evaluation of high-resolution 3D ultrasonography, *in-vivo* micro-CT and 9.4T MRI

**DOI:** 10.1038/s41598-017-14759-1

**Published:** 2017-10-27

**Authors:** Johannes Linxweiler, Christina Körbel, Andreas Müller, Eva Jüngel, Roman Blaheta, Joana Heinzelmann, Michael Stöckle, Kerstin Junker, Michael D. Menger, Matthias Saar

**Affiliations:** 1 Department of Urology, Saarland University, Homburg/Saar, Germany; 2 Institute for Clinical and Experimental Surgery, Saarland University, Homburg/Saar, Germany; 3 Department of Diagnostic and Interventional Radiology, Saarland University, Homburg/Saar, Germany; 4 Department of Urology, Mainz University Medical Center, Mainz, Germany; 5 Department of Urology, Frankfurt University Medical Center, Frankfurt/Main, Germany

## Abstract

In this study, we aimed to comparatively evaluate high-resolution 3D ultrasonography (hrUS), *in-vivo* micro-CT (μCT) and 9.4T MRI for the monitoring of tumor growth in an orthotopic renal cell carcinoma (RCC) xenograft model since there is a lack of validated, non-invasive imaging tools for this purpose. 1 × 10^6^ Caki-2 RCC cells were implanted under the renal capsule of 16 immunodeficient mice. Local and systemic tumor growth were monitored by regular hrUS, μCT and MRI examinations. Cells engrafted in all mice and gave rise to exponentially growing, solid tumors. All imaging techniques allowed to detect orthotopic tumors and to precisely calculate their volumes. While tumors appeared homogenously radiolucent in μCT, hrUS and MRI allowed for a better visualization of intratumoral structures and surrounding soft tissue. Examination time was the shortest for hrUS, followed by μCT and MRI. Tumor volumes determined by hrUS, μCT and MRI showed a very good correlation with each other and with caliper measurements at autopsy. 10 animals developed pulmonary metastases being well detectable by μCT and MRI. In conclusion, each technique has specific strengths and weaknesses, so the one(s) best suitable for a specific experiment may be chosen individually.

## Introduction

Orthotopic xenografts are innovative, increasingly used preclinical *in-vivo* models to study renal cell carcinoma (RCC)^1–3^. In these models, cultured human RCC cells or human RCC tissue samples are implanted under the renal capsule of immunodeficient mice using a syringe (cells) or a small capsular incision (tissue). Following successful engraftment, the biological behavior of the tumor (growth rate, local invasion, development of metastases) and its response to various kinds of treatment can be assessed. Compared to subcutaneous (i.e. heterotopic) xenografts, which are still the most frequently used RCC *in-vivo* models, renal subcapsular RCC xenografts are technically demanding but they comprise some crucial advantages concerning a realistic modeling of the situation in humans: The renal subcapsular implantation site far better represents the local microenvironment in which RCCs develop and grow. It is well known, that the organ-specific biological environment can have profound effects on local tumor growth, development of metastases and response to medical treatment^4–6^. Beyond that, the natural routes of local progression and metastatic spread are only realistically displayed when the tumor cells are growing at their original anatomic site. Finally, the renal subcapsular space shows high engraftment rates compared to other implantation sites^7,8^, which is most probably due to its excellent vascularization ensuring a good oxygen and nutrient supply.

However, one important constraint inherent with the use of orthotopic RCC xenografts hampers their widespread adoption, namely the difficulty to detect the tumors and to non-invasively monitor their growth. While this can be easily done in the well-accessible subcutaneous xenografts using a caliper^9^, the non-invasive monitoring of renal subcapsular tumors requires the use of sophisticated imaging techniques. During the last years, imaging techniques commonly used in daily clinical practice for the diagnosis and follow-up of RCC patients have been transferred to the experimental preclinical setting by developing instruments and machines specially designed for the use in rodents and other small animals^10^. These techniques include – among others – ultrasonography (US)^11^, computed tomography (CT)^12^ and magnetic resonance imaging (MRI)^13^. To date there are only very few reports on the use of these innovative imaging techniques in orthotopic xenograft models of RCC. In this study, we therefore evaluated for the first time in direct comparison 3D high-resolution ultrasonography (hrUS), contrast-enhanced *in-vivo* micro-CT (μCT) and 9.4T MRI (MRI) to non-invasively monitor tumor growth in an orthotopic RCC xenograft model.

## Results

### Orthotopic tumor cell engraftment and growth

**Figure 1:**
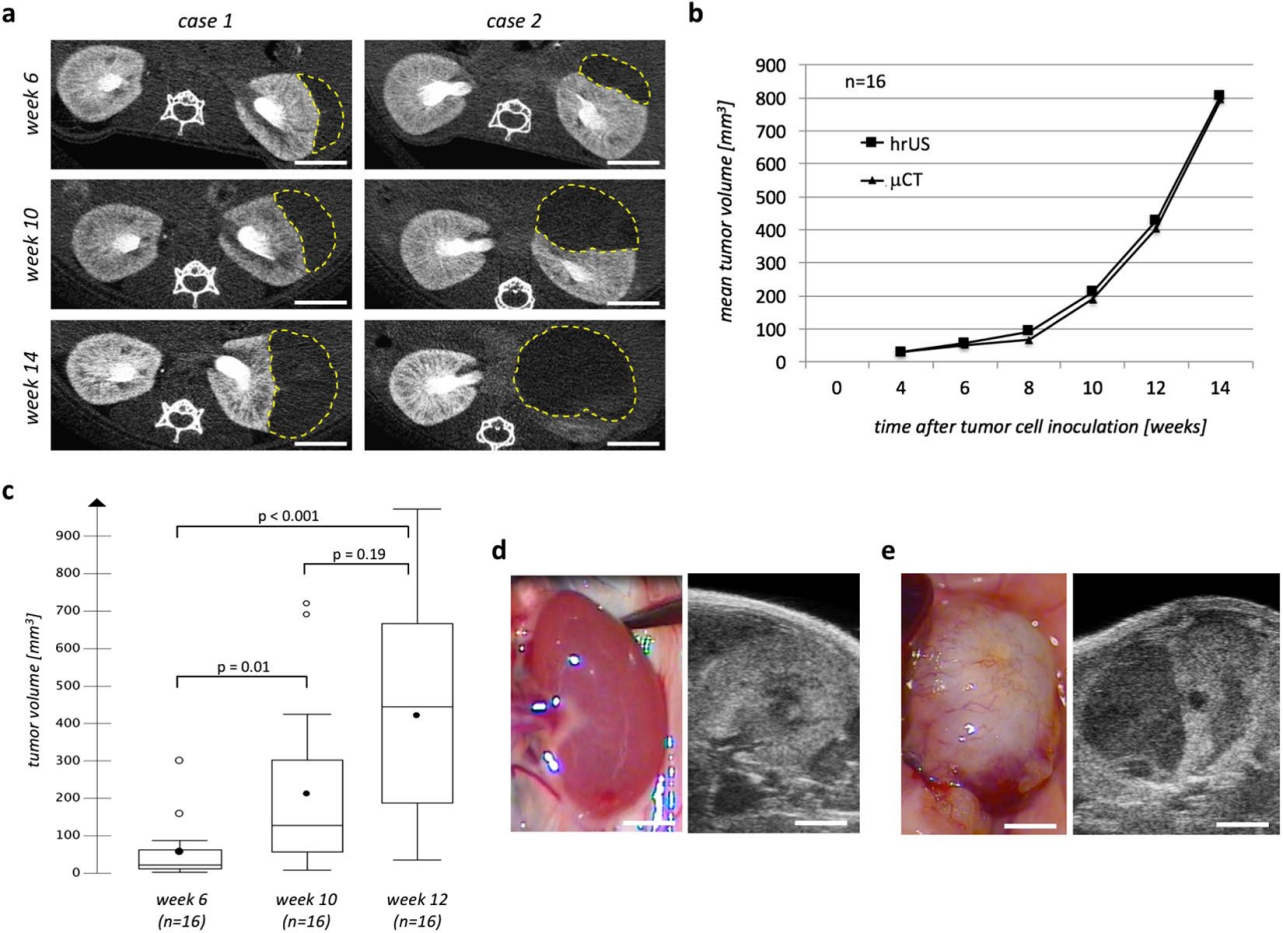
Orthotopic tumor cell engraftment and growth after renal subcapsular implantation of 1 × 10^6^ CAKI-2 cells. (**a**) Representative cross-sectional CT images from two cases 6, 10 and 14 weeks after tumor cell implantation. Tumors are marked by a dashed yellow line. Scale bar = 3 mm. (**b**) Mean tumor volumes as detected by high-resolution ultrasonography (squares) and contrast-enhanced micro-CT (triangles) 4, 6, 8, 10, 12 and 14 weeks after orthotopic tumor cell implantation. (**c**) Box-Whisker plots of tumor volumes 6, 10 and 12 weeks after tumor cell implantation as detected by ultrasonography. Each box represents the range from the first quartile to the third quartile. The median is indicated by a line, the mean by a black circle. Outliers outside the 1.5 fold interquartile range are displayed by white circles. (**d**) Microphotograph (left) and hrUS-image in transverse orientation (right) of a normal left kidney of a 22 weeks old female Balbc/nude mouse. Scale bar = 3 mm. (**e**) Microphotograph (left) and hrUS-image in transverse orientation (right) of a tumor-bearing left kidney in a 22 weeks old female Balbc/nude mouse 14 weeks after renal subcapsular implantation of 1 × 10^6^ CAKI-2 cells. Scale bar = 3 mm.

**Figure 2:**
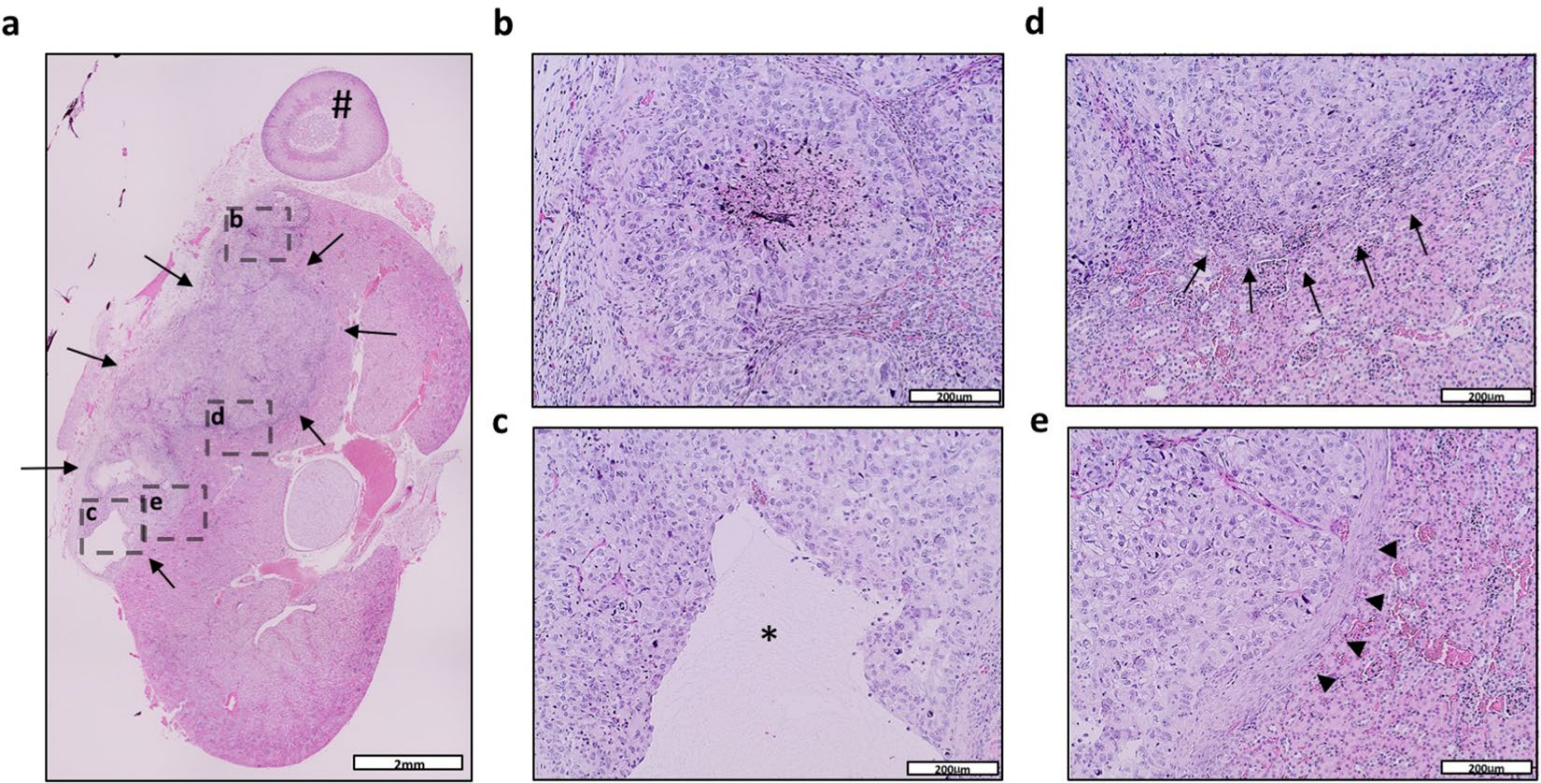
Histologic evaluation (H&E staining) of orthotopic tumors after autopsy. (**a**) Representative microphotograph of a tumor-bearing kidney. The tumor is marked by arrows, the adrenal gland by a rhomb (#). Scale bar = 2 mm. (**b**–**e**) Details from (**a**) showing central necrosis (**b**), malignant cyst formation (**c**, asterisk), invasive growth pattern (**d**, arrows) and fibrotic pseudocapsule (**e**, arrowheads), respectively. Scale bar = 200 μm

Complications during the surgical procedure, such as perforation of the renal capsule or excessive subcapsular bleedings were not observed. Slight recoil of the tumor cell suspension was observed in four cases despite compression of the injection site with a cotton swab for one minute. None of the animals died during surgery or during the early postoperative course. Four weeks after tumor cell inoculation orthotopic tumors, visualized by hrUS (Fig. 1d,e) and μCT (Fig. 1a), were observed in all animals. During the 14 weeks of follow-up, these tumors showed an exponential growth curve (Fig. 1b). Nonetheless, the tumor volumes measured at the single time points of postoperative imaging showed a quite high variability (Fig. 1c). At autopsy, solid hypervascularized masses with a pale yellow to white surface were seen at the site of tumor cell inoculation (Fig. 1d,e). Histological evaluation after autopsy revealed solid tumors (Fig. 2a), occasionally showing central necrosis (Fig. 2b) or cyst formation (Fig. 2c). At the tumor margin cancer cells mostly showed an infiltrative growth pattern (Fig. 2d) although in some cases a fibrotic pseudocapsule could be seen (Fig. 2e). Rarely – as depicted in Fig. 2 - these two features were even seen within the same tumor.

### Imaging properties

Comparison of the three tested imaging techniques revealed profound differences in imaging properties and spatial resolution: HrUS images provided an excellent resolution (30 μm) with a slice thickness of 100 μm (step size of ultrasound linear motor). For μCT, we used a protocol providing a spatial resolution of 18 μm, because we considered this to be sufficient to precisely calculate tumor volumes and to visualize the renal tumor and the surrounding soft tissue. However, using other protocols, the resolution could be further increased to 9 μm. However, such a protocol is associated with a much higher radiation exposure and a longer examination time. MRI showed the lowest spatial resolution (100 μm), the slice thickness was adjusted to 700 μm to avoid an excessive duration of the examination.

**Figure 3:**
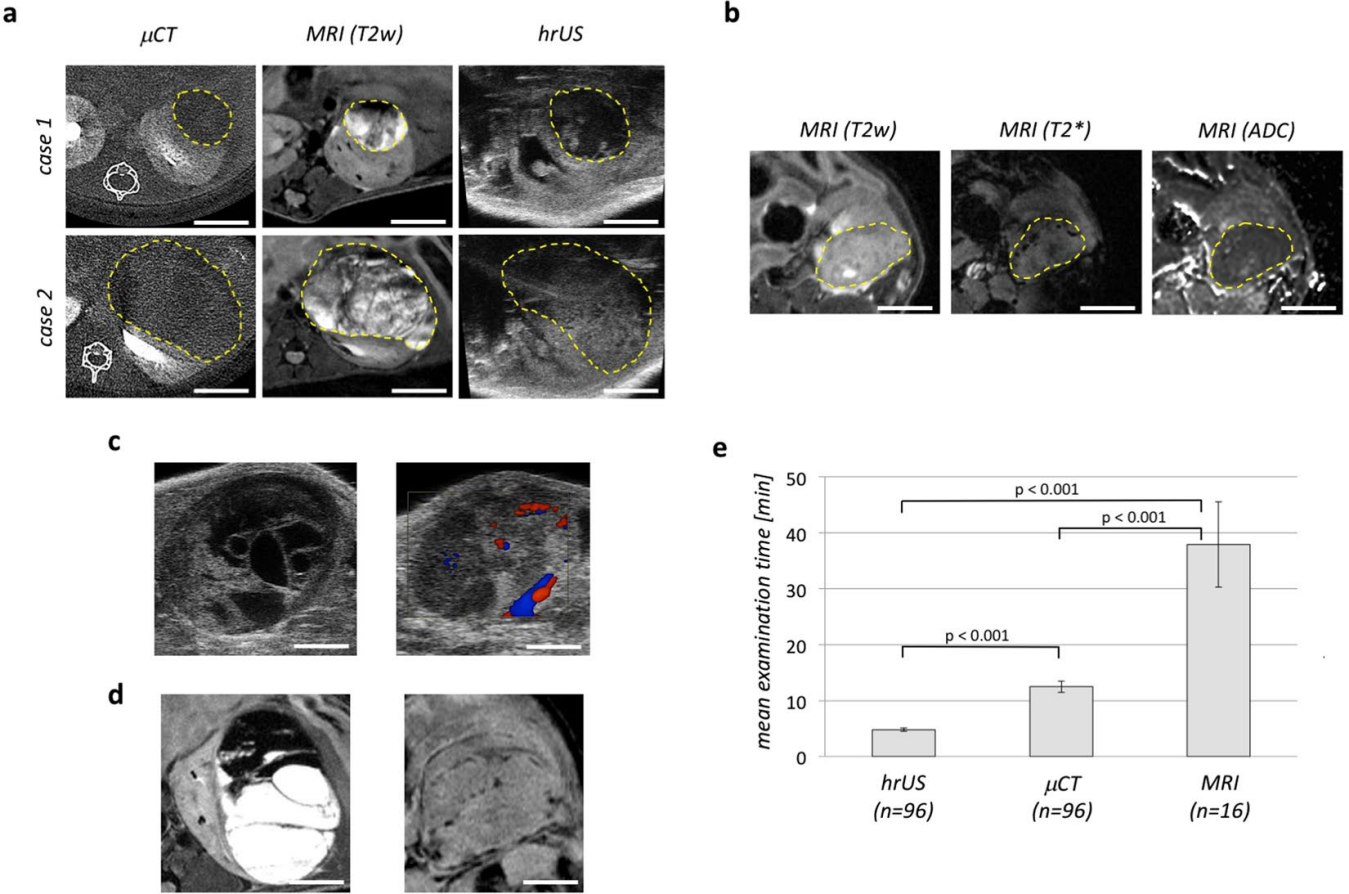
Radiographic imaging properties and examination time. (**a**) Contrast-enhanced μCT (left), T2-weighted MRI (middle) and hrUS images (right) from two representative cases at week 6 (case 1) and week 14 (case 2) after renal subcapsular tumor cell implantation. Tumors are marked by a dashed yellow line. Scale bar = 3 mm. (**b**) MRI-images from a representative case at T2-weighted (left), T2*- (middle) and diffusion-weighted sequences (right). Tumors are marked by a dashed yellow line. Scale bar = 3 mm. (**c**) hrUS images showing a partially cystic tumor (left, B-mode) and tumor- and kidney associated vasculature (right, duplex-mode). (**d**) MRI images of a cystic tumor (left, T2-weighted) and of tumor-associated vasculature (right, T2*-weighted, dark signals). (**e**) Mean examination time for hrUS, μCT and MRI. Error bars indicate standard deviation.

Image analysis revealed that hrUS provides a good soft tissue contrast and enables a reliable identification and delineation of the tumor margins (Fig. 3a). Cystic and solid tumor components as well as intratumoral bleedings could be reliably differentiated (Fig. 3c). Besides that, with the use of the duplex or power Doppler modes it is possible to visualize tumor-associated vasculature (Fig. 3c). In μCT images renal tumors appeared homogenously radiolucent (Fig. 3a). Solid and cystic parts as well as intratumoral bleedings could hardly be differentiated. When acquiring images immediately after intravenous bolus injection of the contrast agent, a signal enhancement could be observed in the renal parenchyma, the renal pelvis and the ureter. In contrast, no contrast agent uptake by the tumor itself was seen. A visualization of the renal vessels or the tumor-associated vasculature was not possible using μCT. MRI (Fig. 3b) provided the best soft tissue contrast of all three imaging techniques. Intratumoral as well as surrounding soft tissue could be visualized in detail. Intratumoral cysts could be clearly identified as rounded, thin-walled structures with intense signals in T2-weighted sequences (Fig. 3d left). Beyond that, intratumoral bleedings and larger blood vessels at the tumor margin were clearly visible as dark signals in T2*-weighted sequences (Fig. 3d right). Diffusion-weighted images showed a marked diffusion restriction (dark signals in ADC maps) as it is to be expected for cell-rich tissue (i.e. tumors).

### Examination time

Mean examination times were 4.8+/−0.4 minutes for hrUS (n = 96), 12.5+/−0.9 minutes for μCT (n = 96) and 37.9+/−7.8 minutes for MRI (n = 14) (Fig. 3c). Examination times were defined as the time span from induction of anesthesia until the end of the examination. All procedures could be performed in isoflurane inhalation anesthesia with continuous monitoring of body temperature and breathing rate.

### Tumor volumetry

**Figure 4:**
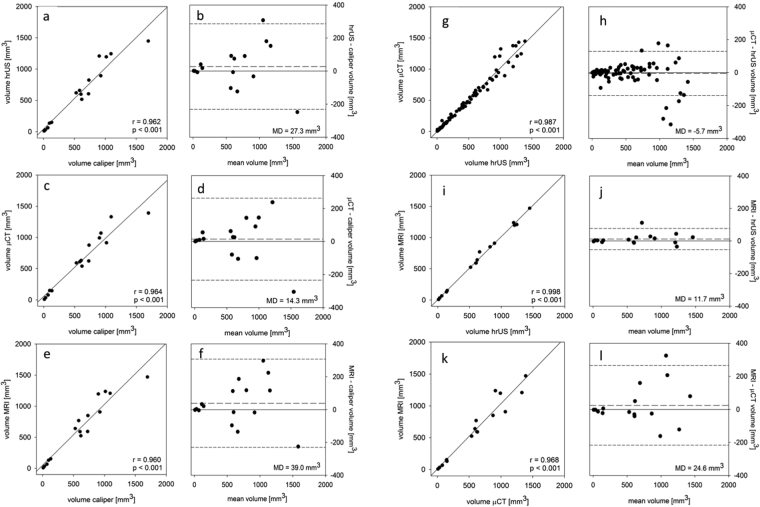
Primary tumor volumetry. Correlation of primary tumor volumetry by caliper measurement (n = 16), hrUS (n = 96), μCT (n = 96) and 9.4T MRI (n = 16), as assessed by linear correlation analysis (**a**,**c**,**e**,**g**,**i**,**k**) and Bland-Altman analysis (**b**,**d**,**f**,**h**,**j**,**l**). To create a Bland-Altman plot, the mean for primary tumor volumes measured by the two methods to be compared was plotted on the horizontal axis and the differences between the two types of measurement were plotted on the vertical axis. long-dashed line = median deviation (MD), short-dashed line = double standard deviation, r = correlation coefficient.

Tumor margins could be well delineated with all three imaging modalities and then be used for calculation of primary tumor volume. Figure 4 shows a consistency analysis of tumor volumes determined with hrUS (n = 96), μCT (n = 96) and MRI (n = 16) by linear correlation analysis (Fig. 4a,c,e,g,i,k) and Bland-Altman analysis (Fig. 4b,d,f,h,j,l). As a further comparator, tumor volumes were determined by caliper measurement at the time of autopsy. In all cases, correlation coefficients were > 0.96 indicating a very good correlation of all applied imaging techniques. Across all groups, variation of calculated volumes increased with higher tumor volumes. Besides that, correlation coefficients were slightly lower when caliper measurement was compared to one of the other three techniques.

### Development and Detection of Metastases

**Figure 5:**
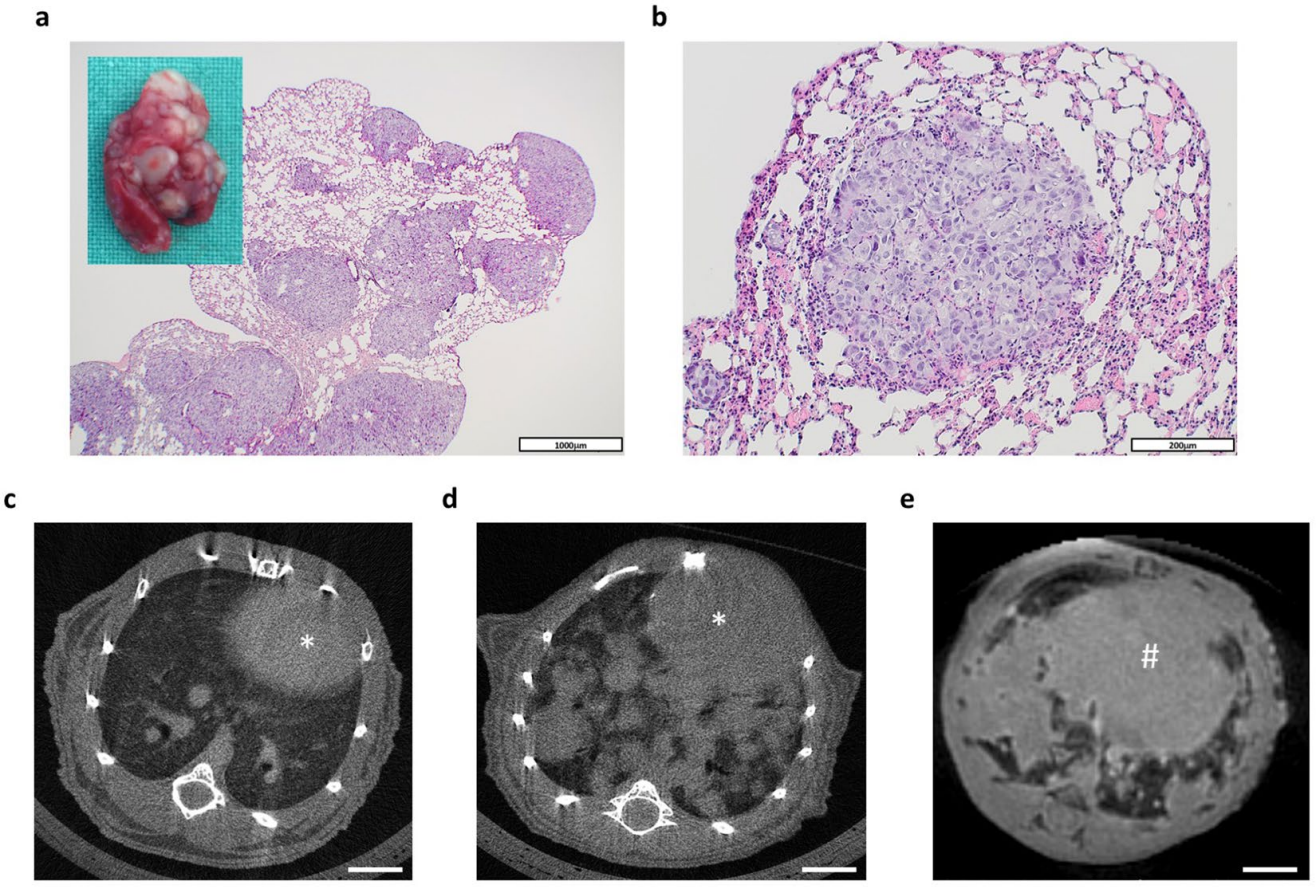
Development and detection of pulmonary metastases. (**a**) Representative microphotograph and macroscopic appearance (insert) of a lung bearing multiple metastases. Scale bar = 1000 μm. (**b**) Representative microphotograph of a single pulmonary metastasis. Scale bar = 200 μm. (**c**,**d**) Representative cross-sectional μCT images of a healthy lung (**c**) and a lung with multiple metastases (**d**). The heart is indicated by an asterisk (*). (**e**) Representative cross-sectional MRI-image (UTE, ultrashort echo time) of the basal parts of a lung with multiple metastases. The diaphragm is indicated by a rhomb (#). Scale bars = 3 mm.

At autopsy 14 weeks after renal subcapsular tumor cell inoculation, multiple pulmonary metastases could be observed in 10 of 16 animals (Fig. 5a,b). These metastases were well detectable by μCT (Fig. 5c,d) using a resolution of 18 μm and the breathing gating function. Their visualization was also possible using MRI (UTE, ultrashort echo time), though with a markedly lower resolution and longer examination time (Fig. 5e). Due to the inherent inability of ultrasound waves to pass air, hrUS could not serve for the detection of pulmonary metastases. Lymphonodal, visceral or skeletal metastases were seen neither macroscopically nor histologically.

## Discussion

In this study we showed that high-resolution 3D ultrasonography, contrast-enhanced *in-vivo* micro-CT and 9.4T small animal MRI are feasible tools for an accurate, non-invasive monitoring of tumor growth in orthotopic RCC xenograft models. All three techniques, which were for the first time evaluated in direct comparison here, were able to reliably measure primary tumor volume and showed a very close correlation with each other as well as with caliper measurements at autopsy. However, they differ considerably concerning technical aspects and the information they can provide beyond primary tumor volume.

HrUS allows for a very fast assessment of the primary tumor with an excellent resolution. A further advantage is the lack of exposure to ionizing radiation. Thus, there is no limitation concerning the number of examinations performed during an experiment. Besides the analysis of primary tumor volume and location, information about its tissue composition – like cystic and solid parts - or intratumoral bleedings can be reliably acquired [10, own data]. In contrast, the visualization of pulmonary or skeletal metastases is not possible. Lymph node or visceral organ metastases were not observed in our study, however their detection with hrUS seems to be feasible^14,15^. Beyond that, contrast-enhanced ultrasound imaging using “naked” or antibody-/ligand-conjugated microbubbles might be able to even better visualize orthotopic tumors as well as associated angiogenesis^16^ and potential treatment effects^17^. A drawback of the hrUS technique is that the interpretation of the acquired images requires some expertise, yet this is also true for μCT and MRI. However, this drawback can easily be overcome. In our experience, even people without any ultrasonography expertise can be trained sufficiently within a few hours to reliably identify tumors. Noteworthy, proper positioning of the animals is a key for achieving good image quality in all three tested modalities. When primary tumor growth is of central interest, hrUS would be our method of choice due to its very short examination time, ease of use, no need to use contrast agents and lack of radiation exposure. Furthermore, the costs for purchase and maintenance of a small animal high resolution ultrasound system are moderate when compared, for example, with MRI.

Micro-CT is able to visualize orthotopic tumors as homogenously radiolucent masses, which can be easily delineated from healthy kidney tissue when the contrast agent is injected as a bolus immediately before image acquisition. In our hands, the administration of conventional iodine contrast medium (300 mg iodine/ml; 0.5 μl/g body weight) as used in daily clinical practice for humans proved to be safe. Of note, the contrast medium was already in its excretory phase when acquiring CT images immediately after bolus injection. Therefore, when arterial phase imaging is of interest – for example for the analysis of tumor-associated vasculature - the use of an infusion pump system is necessary^18^. Alternatively, modern nanoparticles or high molecular weight polymers might be used which stay in the circulation for several days^19,20^. Micro-CT provides an excellent resolution with pixel sizes of 9, 18 or 35 μm - thereby outperforming hrUS and MRI by far in this regard. In our experiments we used a resolution of 18 μm as this provides sufficient information for a precise analysis of primary tumor volume and keeps the examination time under 15 minutes when imaging the whole thorax and abdomen at once. If the kidneys or the primary tumor are the only focus of interest, the examination time can be as short as 5 to 6 minutes. In this study we could further show that pulmonary metastases can be excellently visualized and monitored by μCT. Bone metastases were not observed, but might be detectable equally well with this technique^21^. The detection of visceral and lymph node metastases with μCT is substantially limited by its relatively poor soft tissue contrast. However, this poor contrast can be markedly enhanced with the use of modern, nanoparticle-based contrast agents, which might even enable the detection of lymph node metastases^22^. Another point to consider with this technique is the exposure to ionizing radiation, which can vary considerably depending on experimental settings (resolution, filter, body area scanned, X-ray voltage etc.). Using our adjustments as specified in the materials and methods section, up to six CT scans per animal are possible until the maximum allowed radiation dose is reached^12^.

Small animal MRI at 9,4T provides an excellent visualization of intra- and peritumoral soft tissues without exposure to ionizing radiation. While T2-weighted images proved to be best suited for morphological evaluation (tumor volume, presence of metastases), additional modalities like blood oxygenation level dependent (BOLD), T2*, arterial spin labeling (ASL), dynamic contrast-enhanced (DCE) MRI or diffusion-weighted imaging (DWI) can further provide information about tumor biology. BOLD-MRI has been shown to facilitate discrimination of RCC from benign renal masses and grading of clear-cell RCC in patients^23^. Furthermore, measurements of renal tumor oxygenation have been performed with this method in mice^24^. In rats, ASL has been used to investigate acute kidney injury induced by intravenous application of x-ray contrast agent^25^. Using DCE- and DWI-MRI, Jeon and colleagues could show that small animal MRI might also be able to assess early therapeutic response to small molecule tyrosine kinase inhibitors in subcutaneous RCC xenograft models^26^. Beyond that, it is also possible to screen for pulmonary, visceral or lymphonodal metastases^27,28^ and to monitor renal function^29,30^. However, the main restrictions of MRI imaging are its relatively poor spatial resolution (compared to hrUS and μCT) as well as the fact that it is quite time consuming. In our study, the mean examination time with MRI was over 30 minutes. Notably, we always acquired three different datasets during one imaging study (T2, T2*, ADC). When only T2-weighted images are acquired, which is sufficient for morphological evaluation and tumor volume determination, the examination time can be reduced to about 15 to 20 minutes. Finally, the widespread use of experimental MRI systems might be limited as they are very expensive both to purchase and to operate.

Caliper measurements and bioluminescence are currently the two most commonly used methods to track tumor growth in RCC xenograft models^31,32^. However, despite being well established and widely used, these techniques come along with some serious constraints. Measurement of tumor size with a caliper is non-invasive, quick and easy to perform. However, this method can only be used repeatedly in subcutaneous xenograft models in which the tumors are directly accessible from outside. In our view, these kinds of heterotopic xenografts are not an ideal way to model RCC in preclinical *in-vivo* studies since we consider them to be inferior to orthotopic (renal subcapsular) xenografts for several reasons as further delineated in the introductory section of this article. Moreover, tumor volumes have to be calculated from tumor length, width and height using an ellipsoid volume formula, which is much less accurate compared to the imaging methods tested in our study in whom tumor volume is calculated from many cross-sectional images.

Bioluminescence is a commonly used and sensitive method to screen for metastases in xenograft models of RCC and other tumors. Yet, it depends on the stable transfection of tumor cells with luciferase-encoding plasmids, which is quite easy to perform but might already lead to alterations in gene expression and tumor cell biology. In addition, this method is restricted to the use of immortalized cell lines amenable to luciferase transfection and cannot be regularly applied yet when patient-derived tumor tissue is used like in modern PDX models^1,33^. Another drawback of bioluminescence is the fact that a serial quantification of primary tumor volume or the volume of particular metastases at different examination time points can only be done roughly using the intensity of luminescence signals in two dimensional view. Moreover, neither biological nor morphological information about tumor tissue (cystic or solid parts, intratumoral bleedings, infiltration of adjacent organs, central necrosis) can be obtained as it is possible with the imaging techniques used in this study.

Considering all these points, hrUS, μCT and MRI represent attractive alternatives to the methods commonly used to monitor tumor burden in orthotopic RCC xenograft models. Besides established, immortalized cell lines – as in our study – primary RCC cell lines^34^ or even intact tumor tissue^2^ can be used in these models thereby yielding an even more individualized, patient-derived approach. Here, our study is limited by the fact that we only used one well-established RCC cell line for orthotopic tumor induction, but data on primary cell cultures as well as grafted tumor tissue are on their way.

Taken together, each of the three imaging techniques tested in our study entails its individual strengths and weaknesses (summarized in Table 1). Therefore, the method best suitable to answer a particular scientific question using orthotopic RCC xenografts should be chosen individually. When primary tumor growth is of major interest, hrUS represents a fast and reliable option. If screening for metastases or a better soft tissue contrast are needed, μCT or MRI might be more appropriate, respectively. However, as we could show in our study, it is also possible to use two or even all of these imaging techniques in one experimental series.

**Table 1:** Individual strengths and weaknesses of the tested imaging techniques for monitoring of local and systemic tumor burden in orthotopic RCC xenograft models. hrUS = high-resolution ultrasonography, μCT = contrast-enhanced *in-vivo* computed tomography, MRI = 9.4T magnetic resonance imaging, PET = positron emission tomography, BOLD = blood oxygen level dependent, ASL = arterial spin labeling, DCE = dynamic contrast-enhanced, DWI = diffusion-weighted imaging.

	hrUS	μCT	9.4T MRI
spatial resolution	very high (33 μm)	very high (18 μm)	high (100 μm)
soft tissue contrast	good	poor	very good
metastases screening	liver	bone, lung	all organ systems
options for functional imaging	Doppler^35^, contrast agents^16,17,36^, photoacoustic imaging^37^	contrast agents^20^, combination with PET^38^	alternative protocols (BOLD, ASL, DCE, DWI)^23–30^, contrast agents^17,39^, combination with PET^38^
examination time	very short (5 minutes)	short (12.5 minutes)	long (37.9 minutes)
radiation exposure	none	yes (radiation dose depending on protocol and number of scans)	none
costs (purchase)	moderate	moderate	high

## Methods

### Animals

Female athymic nude mice (Balb/c nude, CAnN.Cg-*Foxn1* ^*nu*^/Crl, 8–10 weeks old, Charles River Laboratories, Sulzfeld, Germany) were kept in isolated ventilated cages under specific pathogen-free conditions in a temperature- and humidity-controlled 12 hour dark/light environment at the animal care facility of the Institute for Clinical and Experimental Surgery at Saarland University. Animals had free access to tap water and standard pellet food and their health status was monitored daily. All experiments were approved by the local governmental animal care committee (Landesamt für Verbraucherschutz des Saarlandes; Approval No. 28/2014) and conducted in accordance with the German legislation on protection of animals and the National Institutes of Health Guide for the Care und Use of Laboratory Animals (NIH Publication #85–23 Rev. 1985).

### Cell culture

CAKI-2 human renal cell carcinoma cells were cultured at 37 °C in RPMI 1640 medium (Sigma Aldrich, St. Louis, USA) containing 10% fetal bovine serum (FBS) (Biochrom, Berlin, Germany) in a humidified environment with 5% CO_2_. The identity of CAKI-2 cells was proven by STR fingerprinting and RFLP. For renal subcapsular inoculation the cells were harvested at 70–80% confluence, counted and suspended in a 1:3 mixture of Matrigel (Corning, New York, USA) and culture medium at a density of 1 × 10^6^ cells/10 μl. This suspension was kept on ice until it was transferred to a cooled 10 μl Hamilton syringe (Hamilton, Reno, USA) immediately before renal subcapsular injection.

### Orthotopic tumor cell implantation and follow-up

Renal subcapsular tumor cell implantation was performed under intraperitoneally applied anesthesia (75 mg/kg Ketamin, 15 mg/kg Xylazin) using a stereo-microscope (Leica M651; Leica Microsystems AG, Heerbrugg, Switzerland). Animals were placed in a lateral position (right side down) and the skin was opened by a 5–7 mm flank incision parallel to and below the 13^th^ rib. After dissection of the subcutaneous and muscle tissue, the left kidney was pushed out of the incision by applying gentle pressure with two fingers. Then, 10 μl of the Matrigel®/medium suspension containing 1 × 10^6^ CAKI-2 cells were inoculated under the left renal capsule using the Hamilton syringe. For this, the renal capsule was penetrated with the needle at the lower kidney pole. The needle was then pushed forward under the capsule towards the upper kidney pole for 5–6 mm and, finally, the tumor cell suspension was slowly injected. After removing the needle, gentle pressure was applied to the injection site for 60 seconds using a moistened cotton swab to avoid recoiling of the tumor cell suspension. The muscle layer and the skin were closed using 5/0 Vicryl rapide absorbable suture material (Ethicon, Somerville, USA) in a single knot technique. At the end of the procedure, mice received a subcutaneous injection of 20 mg/kg Tramadol for pain relief. To follow up orthotopic tumor engraftment and growth as well as the development of metastases, the mice underwent hrUS and μCT examinations 4, 6, 8, 10, 12 and 14 weeks after renal subcapsular tumor cell implantation. Additionally, a MRI was performed at 14 weeks. Mice were sacrificed after 14 weeks by cervical dislocation. During autopsy, primary tumor volume was further determined using a caliper and abdominal visceral organs, lungs and lymph nodes were inspected for macroscopically visible metastases. Primary tumor tissue, lower extremity bones, lungs, liver, spleen and renal and lumbar aortic lymph nodes were harvested for histological evaluation.

### High resolution 3D ultrasonography (hrUS)

Ultrasonography of the primary tumor was performed under isoflurane inhalation anesthesia (induction: 4%, maintenance: 2%; flow 2 l/min) using a VisualSonics Vevo 770 small animal ultrasonography system (FUJIFILM VisualSonics Inc., Toronto, Canada). Animals were fixed in a prone position on a heated stage providing temperature feedback and heart rate monitoring (THM-100; Indus Instruments, Houston, USA). After the left kidney was centered in the focus, the 3D acquisition mode was started. The 30 MHz ultrasound probe RMV 707b (FUJIFILM VisualSonics Inc., Toronto, Canada) scanned across the mouse skin and acquired two-dimensional images of the left kidney in axial/transverse orientation at regular spatial 100 μm intervals. A predefined parallel geometry of the 2D images allowed fast 3D image reconstruction^38^. Tumors were identified and marked in the acquired cross-sectional 2D images and tumor volumes were calculated by the volumetric analysis function of the ultrasound software (Vevo Lab; FUJIFILM VisualSonics Inc.) from multiple marked tumor perimeters.

### Contrast-enhanced *in-vivo* micro CT (μCT)

Contrast-enhanced *in-vivo* micro CT was performed under isoflurane inhalation anesthesia using a Bruker Skyscan 1176 system (Bruker Corporation, Billerica, USA). Immediately after induction of anesthesia, iodine contrast medium (Accupaque 300, GE healthcare, Chalfont St. Giles, UK; 300 mg iodine/ml, 0.5 μl/g body weight) was injected intravenously into the retrobulbar space. Thereafter, mice were fixed in supine position on a carbon fibre examination bed. After performing an overview scan of the whole animal and defining the region of interest, the actual CT scan was started using the following experimental setups: Aluminium 0.5 mm filter, Averaging 2, 180° scanning, 0.7° steps, 18 μm resolution, exposure time 220 ms per image (with 304 images acquired per scan), source current 497 μA, source voltage 50 kV. To enable the visualization of the primary tumor as well as the detection of pulmonary or skeletal metastases, the body area scanned included the whole thorax, abdomen and pelvis (oversize scan). Cross-sectional CT images were reconstructed from the acquired radiographs with the “NRecon” Reconstruction software (Bruker Corporation, Billerica, USA) using established protocols^39^. For tumor volume measurement, the “CT An” software (Bruker Corporation, Billerica, USA) was used according to the manufacturers instructions.

### 9.4T small animal MRI (MRI)

At 14 weeks after renal subcapsular tumor cell implantation animals were examined in a horizontal-bore, 9.4T MRI animal scanner (Biospec Avance III 94/20; Bruker Biospin GmbH, Ettlingen, Germany) with a BGA12S gradient system (maximum field strength 675 mT m-1, linear inductive rise time 130 µs, maximum slew rate 4673 mT/m/s) run with ParaVision 5.1, with the animals maintained under isoflurane inhalation anesthesia and control of vital parameters as described previously^40^. MRI was performed using a linear polarized coil developed for imaging of the mouse abdomen with an inner diameter of 38 mm (Bruker Biospin GmbH, Ettlingen, Germany). After performing fast low angle shot based 3D localizer for control of animal placement within the isocenter and adjustment of slice geometry, B0 inhomogeneities were compensated by extensive first and second order iterative shimming in eight different three-dimensional orientations. Following shimming Larmor frequency and reference RF pulse strength were readjusted and imaging experiments were started. MRI was performed with prospective triggering, synchronizing imaging experiments to animal respiration via a signal recorded with a pneumatic cushion (Graseby infant respiration sensor; Smiths Medical, Dublin, OH, USA) attached to the abdomen and commercially available software for monitoring of small animals (PC-SAM32; SA Instruments, Inc, Stony Brook, NY, USA). Repetition times (TR), echo times (TE), field of view (FOV), matrix size (MTX), slice thickness (ST), resulting voxel size, number of slices (NS) and number of acquisition (NA) settings for the different MRI sequences employed are given in Supplementary Table S1. T1 and T2* imaging was performed with a 2D multi gradient echo (MGE) sequence with a flip angle of 30°. For T2 weighted imaging a rapid acquisition relaxation enhanced (RARE) protocol with RARE factor of 8, flip back pulse and fat saturation was employed. Diffusion weighted imaging (DWI) for calculation of apparent diffusion coefficient (ADC) maps was established with an echo planar imaging (EPI) sequence with fat saturation as a diffusion trace experiment^41^. To reduce the influence of the number and strength of the diffusion-sensitizing gradients on ADC values, imaging was performed with four different diffusion gradient strengths of 100, 250, 500 and 1000 mT/m in the x, y and z planes. From the twelve independent diffusion experiments and the imaging data acquired without diffusion gradient ADC maps were created by calculating individual consolidated ADC values in mm^2^/s for each voxel sampled after the modified Steiskal-Tanner equation^42–44^ equation as explained in (1) to (3)

1a $${S}_{x}={S}_{0}{e}^{-{\rm{b}}{{\rm{D}}}_{xx}}$$

1b $${S}_{y}={S}_{0}{e}^{-{\rm{b}}{{\rm{D}}}_{yy}}$$

1c $${S}_{z}={S}_{0}{e}^{-{\rm{b}}{{\rm{D}}}_{zz}}$$

2 $${{\rm{S}}}_{DWI}={{\rm{S}}}_{0}{e}^{-{\rm{b}}({{\rm{D}}}_{xx}+{{\rm{D}}}_{yy}+{{\rm{D}}}_{zz})/3}={{\rm{S}}}_{0}{e}^{-{\rm{b}}({{\rm{D}}}_{trace})/3}={{\rm{S}}}_{0}{e}^{-{\rm{b}}{\rm{A}}{\rm{D}}{\rm{C}}}$$

3 $$ADC=-\mathrm{ln}\,({{\rm{S}}}_{DWI}/{{\rm{S}}}_{0})/-{\rm{b}}$$

with b as the apparent magnetic field strength utilized for each voxel. Average ADC were then calculated from the individual ADC values generated from the measurements at the different magnetic field strengths.

### Histology

For histomorphological evaluation the removed organs were fixed in formalin and embedded in paraffin. Sections of 4 μm were cut using a rotation microtome (Leica RM 2125RT; Leica microsystems, Wetzlar, Germany) and mounted on glass slides. Staining was performed using a standard H&E protocol. Bones were decalcified for 48 h in a 14% EDTA-solution after formalin-fixation prior to paraffin-embedding.

### Statistical analyses

Statistical analyses were performed with Excel for Mac 2011 (Microsoft, Redmond, USA), SPSS Statistics Version 23 (IBM, Armonk, USA) and Sigma Plot Version 13 (Systat Software Inc., San Jose, CA, USA). In general, two-tailed statistical tests were performed and p-values < 0.05 were considered statistically significant (alpha-level of 0.05 for all tests used). For comparison of tumor volumes at different time points (Fig. 1c) and examination times of the three imaging techniques (Fig. 3c), a Mann Whitney-U test was used. Correlations of calculated tumor volumes (Fig. 4) were analyzed by linear correlation analysis and Bland-Altman analysis. First, data were tested for normal distribution by a Shapiro-Wilk test. When normally distributed, linear correlation was analyzed by Pearson product moment correlation analysis. When not normally distributed, Spearman rank order correlation analysis was used.

### Data availability

The datasets generated during the current study are available from the corresponding author on reasonable request.

## Electronic supplementary material

Dataset 1
