# Supplementary material for: Experimental imaging in orthotopic renal cell carcinoma xenograft models: comparative evaluation of high-resolution 3D ultrasonography, in-vivo micro-CT and 9.4T MRI
Source: Sci Rep. 2017 Oct 27;7:14249. doi: 10.1038/s41598-017-14759-1 (PMC5660163; doi:10.1038/s41598-017-14759-1)
Supplement: Supplementary file 1 — Dataset 1 [file 41598_2017_14759_MOESM1_ESM.docx]

***Supplementary Information to the article***

*Experimental imaging in orthotopic renal cell carcinoma xenograft models: comparative evaluation of high-resolution 3D ultrasonography, in-vivo micro-CT and 9.4T MRI*

(*Johannes Linxweiler, Christina Körbel, Andreas Müller, Eva Jüngel, Roman Blaheta, Joana Heinzelmann, Michael Stöckle, Kerstin Junker, Michael D. Menger, Matthias Saar)*

**Supplementary table S1**: **Detailed MR imaging sequence parameters.** GRE = gradient recalled echo, MGE = multi gradient echo, RARE = rapid acquisition relaxation enhanced, DWI-EPI = diffusion-weighted imaging echo-planar imaging, FOV = field of view, MTX = matrix size, ST = slice thickness, NS = number of slices, NA = number of averages

| **Sequence** | **Purpose** | **TR**  **(ms)** | **TE (ms)** | **FOV (mm)** | **MTX** | **ST**  **(mm)** | **Voxel Size**  **(µm)** | **NS** | **NA** |
| --- | --- | --- | --- | --- | --- | --- | --- | --- | --- |
| GRE | Localizer | 10.0 | 2.50 | 40x40 | 160x160 | 1 | 250x250x1000 | 3x5 | 1 |
| MGE | Morphology | 225.0 | 2.50  7.0  11.5 | 30x30 | 300x300 | 0.7 | 100x100x700 | 13 | 3 |
| RARE | Morphology | 3000 | 30.0 | 30x30 | 300x300 | 0.7 | 100x100x700 | 29 | 4 |
| DWI-EPI | ADC map | 2750 | 22.5 | 30x30 | 256x256 | 0.7 | 117x117x700 | 11 | 3 |
